# Agri-Food By-Products in Dairy Sector a Review Focused on Phytochemicals, Extraction Methods Health Benefits and Applications

**DOI:** 10.3390/foods15071266

**Published:** 2026-04-07

**Authors:** Roxana Nicoleta Ratu, Florina Stoica, Bianca Andreea Balint, Ionuț Dumitru Veleșcu, Ioana Cristina Crivei, Sebastian-Paul Lucaci, Florin Daniel Lipșa, Gabriela Râpeanu

**Affiliations:** 1 Department of Food Technologies, Faculty of Agriculture, “Ion Ionescu de la Brad” Iași University of Life Sciences, 3 Mihail Sadoveanu Alley, 700489 Iasi, Romania; roxana.ratu@iuls.ro (R.N.R.); bianca.balint@iuls.ro (B.A.B.); ionut.velescu@iuls.ro (I.D.V.); ioana.crivei@iuls.ro (I.C.C.); sebastian.lucaci@iuls.ro (S.-P.L.); 2 Department of Pedotechnics, Faculty of Agriculture, “Ion Ionescu de la Brad” Iași University of Life Sciences, 3 Mihail Sadoveanu Alley, 700489 Iasi, Romania; 3 Department of Food Science, Food Engineering, Biotechnology and Aquaculture, Faculty of Food Science and Engineering, Dunărea de Jos University of Galati, 800201 Galați, Romania; gabriela.rapeanu@ugal.ro

**Keywords:** agri-food by-products, dairy sector, bioactive compounds, green extraction technologies, functional dairy products, health benefits, circular economy

## Abstract

The expansion of the global agri-food industry has led to the generation of large volumes of processing by-products that, although traditionally treated as waste, represent valuable sources of bioactive phytochemicals with potential for sustainable valorisation. This review critically examines the integration of fruit, vegetable, cereal, and dairy processing side streams into functional dairy products. Particular attention is given to recent advances in green and emerging extraction technologies, including ultrasound-assisted extraction, microwave-assisted extraction, and supercritical fluid extraction, with emphasis on their efficiency, environmental performance, and effects on the stability and recovery of phytochemicals. The review also discusses the health-related properties of these bioactive compounds, including antioxidant, anti-inflammatory, and metabolic regulatory effects, in relation to their incorporation into milk, yogurt, cheese, and ice cream matrices. In addition, key barriers to industrial implementation are assessed, including compound stability, sensory constraints, bioavailability, and current regulatory limitations. Beyond direct fortification, the review also considers broader valorisation pathways, such as the biotechnological production of microbial enzymes from agro-industrial biomass, as relevant strategies for supporting circularity. Overall, this review highlights how sustainable extraction approaches and functional dairy innovation can contribute to improving the nutritional value, resource efficiency, and circularity of the dairy sector.

## 1. Introduction

Agri-food processing generates substantial quantities of by-products, including fruit and vegetable pomace, peels, seeds, and dairy residues, which are frequently underutilised despite their significant biochemical potential [1,2,3]. These secondary streams are increasingly recognised not as waste, but as valuable sources of functional compounds. As highlighted by Rațu et al. [4], such materials are particularly rich in dietary fibre, phenolic compounds, carotenoids, and other bioactive phytochemicals, positioning them as promising raw materials for the development of value-added food ingredients. Concurrently, growing environmental and economic pressures to reduce food waste have intensified the interest in sustainable valorisation strategies aligned with circular economy principles [5].

Among these resources, plant-derived by-products have attracted considerable attention due to their high concentrations of bioactive phytochemicals exhibiting antioxidant, antimicrobial, and anti-inflammatory properties. Fernandes et al. [6] reported that fruit and vegetable residues may contain even higher levels of phenolic compounds than their edible counterparts, reinforcing their relevance as functional ingredients. Similar observations have been consistently reported in recent reviews, which emphasise the strong relationship between phytochemical composition and potential health-promoting effects [7,8,9,10]. However, the practical utilisation of these compounds in food systems remains constrained by several factors, including extraction efficiency, chemical stability, and compatibility with complex food matrices.

From a technological standpoint, the recovery of phytochemicals from agri-food by-products has undergone significant advancements. Conventional extraction techniques are increasingly being complemented or replaced by emerging green technologies, such as ultrasound-assisted extraction, microwave-assisted extraction, pulsed electric field-assisted extraction, enzyme-assisted extraction, supercritical fluid extraction, pressurised liquid extraction, and deep eutectic solvents (DES/NaDES), which can improve extraction efficiency while reducing solvent consumption, extraction time, and environmental impact [11,12,13]. Despite these advantages, critical challenges persist, particularly regarding scalability, economic feasibility, process standardisation, and compliance with food-grade requirements, especially when extracts are intended for incorporation into structurally and biochemically complex systems such as dairy products [14,15].

The dairy sector represents a particularly promising application domain for agri-food by-products due to the widespread consumption and nutritional relevance of dairy products. Previous studies have demonstrated that the incorporation of plant-based residues into cheese can significantly enhance antioxidant activity and microbial stability [7,16], while the fortification of fermented dairy products with phenolic-rich extracts has been associated with increased total phenolic content and antioxidant capacity [17,18,19]. Nevertheless, these functional benefits are often accompanied by technological and sensory limitations, including undesirable changes in texture, colour, and flavour, which may compromise consumer acceptance [20,21].

A critical aspect governing the successful integration of these compounds into dairy matrices is the interaction between milk proteins and polyphenols. Such interactions can significantly influence bioaccessibility, antioxidant activity, and physicochemical stability, depending on processing conditions and molecular structure [22]. These effects are particularly pronounced in fermented and ripened dairy products, where ongoing biochemical transformations further modulate matrix behaviour during storage [23]. Therefore, a comprehensive understanding of matrix-level interactions is essential for the rational design of functional dairy systems enriched with agri-food by-products.

Despite the growing body of literature, current reviews remain largely fragmented. Most studies have either approached agri-food by-products from a general food-industry perspective [3,8], or have focused on isolated aspects such as extraction technologies [7,8], phytochemical characterisation [7,9], or specific dairy applications [12,13,14,15]. As a result, there is a lack of integrative frameworks that simultaneously connect phytochemical composition, extraction strategies, biological activity, and technological performance within dairy matrices. In addition, critical aspects such as dose-dependent effects, threshold concentrations, and the balance between functional enhancement and sensory acceptability remain insufficiently addressed.

In this context, the present review aims to provide a comprehensive and critical evaluation of the strategic valorisation of agri-food by-products within the dairy sector. Specifically, this work seeks to: (i) analyse the biochemical profiles of key phytochemical classes present in plant-derived residues and their suitability for dairy fortification; (ii) critically compare conventional and emerging green extraction technologies in terms of efficiency, sustainability, and food-grade applicability; (iii) elucidate the mechanistic links between recovered bioactive compounds and their associated health benefits; and (iv) examine the interactions between by-product-derived compounds and dairy matrices, with particular emphasis on their effects on physicochemical stability, nutritional quality, and sensory properties.

Ultimately, this integrative approach aims to identify current technological bottlenecks and to propose an application-oriented framework supporting the transition towards a circular and resource-efficient dairy industry.

## 2. Novelty and Contribution of This Review

Although the valorisation of agri-food by-products has been extensively discussed in recent years, the existing literature remains largely compartmentalised. Previous reviews have predominantly focused either on general food-industry applications [3], green extraction technologies, phytochemical characterisation and bioactivities, or isolated dairy applications such as cheese or yoghurt fortification [17]. While these contributions provide valuable insights, they seldom integrate the full translational pathway from raw by-product to functional dairy system.

The present review distinguishes itself through a multi-layered integrative framework that simultaneously links the biochemical composition of plant-derived residues, the efficiency and sustainability of extraction and recovery strategies, the mechanistic basis of reported health-promoting effects, and the structural and physicochemical behaviour of recovered compounds within dairy matrices. To further enhance the practical applicability of this review, particular attention is also given to the main factors affecting the scale-up of these approaches, including technological feasibility, economic viability, regulatory considerations, and their integration into existing dairy processing systems.

A key innovative element of this work lies in its matrix-centred perspective. Rather than treating bioactive compounds as isolated functional additives, this review critically analyses their interactions with dairy proteins and lipids, with particular attention to protein–polyphenol binding, bioaccessibility modulation, and structural stability during processing and storage. This approach moves beyond descriptive fortification studies and addresses the mechanistic constraints that ultimately determine industrial feasibility.

Furthermore, this review highlights underexplored yet decisive aspects for practical implementation, including dose-dependent responses, threshold concentrations affecting sensory quality, and trade-offs between functional enhancement and consumer acceptability. By explicitly addressing these variables, the manuscript contributes to bridging the gap between laboratory-scale demonstrations and scalable industrial applications.

Another distinctive contribution is the incorporation of advanced valorisation pathways, such as enzyme production via microbial fermentation on agro-industrial substrates, positioning by-products not only as sources of phytochemicals but also as biotechnological platforms within the dairy processing chain. This systems-level approach aligns molecular innovation with circular economy principles and industrial sustainability objectives.

Collectively, this review provides a structured, application-oriented roadmap for integrating agri-food by-products into dairy systems under technological, regulatory, and market constraints. By synthesising compositional, technological, biological, and industrial dimensions within a unified framework, the present work advances the discourse from general valorisation concepts towards a functional and scalable dairy-specific strategy.

## 3. Methodology

**Figure 1:**
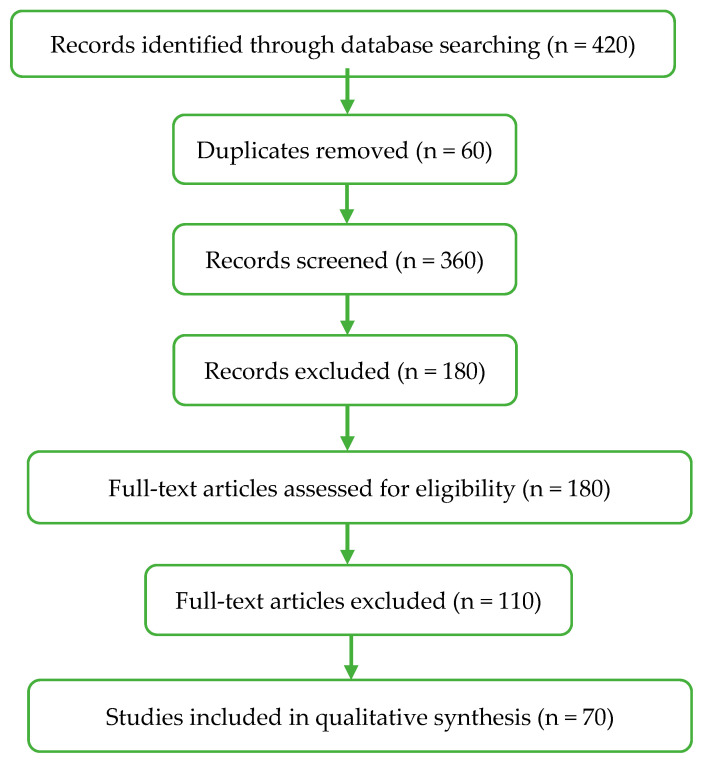
PRISMA-informed flow diagram illustrating the literature search, screening, eligibility assessment, and study selection process applied in this review.

The present review was conducted as a structured, PRISMA-informed literature review designed to provide a comprehensive and critical synthesis of current knowledge on the valorisation of agri-food by-products in dairy systems [24]. The methodology was developed to ensure transparent study identification, screening, and selection, thereby improving reproducibility and reducing selection bias. The literature selection process is summarised in Figure 1.

### 3.1. Search Strategy and Data Sources

A multi-stage literature search was performed using major scientific databases, including Web of Science, Scopus, ScienceDirect, and PubMed. The search strategy was designed to capture recent and relevant advances in the recovery and application of bioactive compounds from agri-food by-products in dairy systems.

The search was restricted to publications from the last 10–15 years (with emphasis on studies published after 2015) to reflect current technological developments and emerging research trends. A combination of Boolean operators (AND/OR) and targeted keywords was applied, including: “agri-food by-products”, “food waste valorisation”, “bioactive compounds”, “polyphenols”, “green extraction technologies”, “ultrasound-assisted extraction”, “microwave-assisted extraction”, “supercritical fluid extraction”, “functional dairy products”, “dairy fortification”, and “circular economy”.

To enhance coverage and minimise omission of relevant studies, reference lists of selected articles were also screened manually (backward search).

### 3.2. Study Selection and Eligibility Criteria

The selection process followed a stepwise screening approach based on relevance, scientific quality, and applicability to the scope of the review.

Studies were included if they met the following criteria:

(i) Published in peer-reviewed journals;
(ii) Written in English;
(iii) Reported experimental or review data on agri-food by-products as sources of bioactive compounds;
(iv) Included quantitative or semi-quantitative data on phytochemical composition, extraction efficiency, or functional properties;
(v) Demonstrated relevance to dairy applications, including milk, yoghurt, cheese, ice cream, or whey-based systems.

Studies were excluded if they:

(i) Lacked experimental validation or contained insufficient methodological detail;
(ii) Focused exclusively on non-food applications (e.g., biofuels, materials);
(iii) Did not establish a clear link between by-product valorisation and functional or technological outcomes in food systems;
(iv) Were duplicates or preliminary reports without full data availability.

The initial search yielded a total of 420 records, which were subsequently filtered based on title and abstract screening. Following duplicate removal and eligibility assessment through full-text evaluation, 70 studies were ultimately included in the final qualitative synthesis. The initial search yielded a total of 420 records, which were subsequently filtered based on title and abstract screening. Following duplicate removal and eligibility assessment through full-text evaluation, 70 studies were ultimately included in the final qualitative synthesis. The selection process is illustrated in Figure 1. It should be noted that the total number of references cited in this review (*n* = 115) also includes additional relevant sources used for contextualisation, theoretical background, and discussion, which were not part of the core dataset subjected to the structured selection process.

### 3.3. Data Extraction and Synthesis

Relevant data were extracted and categorised according to:

(i) Type and origin of agri-food by-product;
(ii) Class of bioactive compounds (e.g., polyphenols, carotenoids, dietary fibres);
(iii) Extraction and recovery technologies;
(iv) Reported yields and functional properties;
(v) Application in dairy matrices;
(vi) Technological, sensory, and nutritional outcomes.

The collected information was synthesised using a comparative and thematic approach, enabling the identification of relationships between extraction methods, compound stability, and functional performance in dairy systems.

Particular emphasis was placed on:

(i) The impact of processing conditions on the stability of thermolabile compounds;
(ii) Interactions between bioactive compounds and dairy matrices (e.g., protein–polyphenol interactions);
(iii) Technological constraints related to industrial scalability;
(iv) Sensory implications and consumer acceptance.

This integrative analysis allowed the identification of current research gaps and facilitated the development of an application-oriented framework linking extraction strategies to functional dairy product design.

## 4. Classification and Industrial Origin of Agri-Food Side Streams

**Figure 2:**
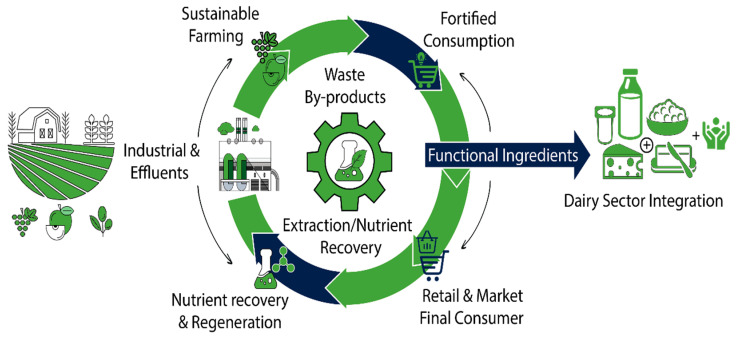
The circular agri-food by-product economy.

The transition towards sustainable food systems increasingly relies on the redefinition of agro-industrial residues as secondary raw materials rather than waste streams [25]. In the context of intensified global food production, these by-products represent a dual challenge: they contribute significantly to environmental burdens while simultaneously offering a largely untapped reservoir of functional compounds with high industrial value [26]. Within the dairy sector, their integration follows a circular and resource-efficient model, in which raw side streams are transformed into functional ingredients through targeted processing strategies, as conceptually illustrated in Figure 2.

Figure 2 illustrates the circular transition from primary production to by-product generation, recovery, and reintegration into dairy systems, highlighting the key stages where value can be reintroduced into the food chain.

Importantly, not all by-products exhibit equivalent suitability for dairy applications. Their technological relevance is primarily determined by compositional factors (e.g., fibre content, polyphenolic profile, lipid fraction) and their compatibility with dairy matrices. Consequently, a systematic classification based on origin and functional composition is essential for guiding their efficient utilisation in dairy systems.

### 4.1. Botanical and Cereal-Based Residues

Fruit and vegetable processing generates a heterogeneous biomass, including pomace, peels, seeds, and residual tissues, which may account for up to 50% of the initial raw material [27]. These residues are particularly relevant for dairy applications due to their high concentration of bioactive compounds and their multifunctional technological roles.

For example, apple pomace—representing approximately 25% of the fruit mass—is a rich source of pectin and phenolic compounds, making it highly suitable for improving water-holding capacity and reducing syneresis in fermented dairy products [28,29]. Similarly, tomato processing residues concentrate lycopene in peel fractions at significantly higher levels than in edible tissues, providing a natural source of lipophilic antioxidants and pigments for dairy fortification [30].

**Figure 3:**
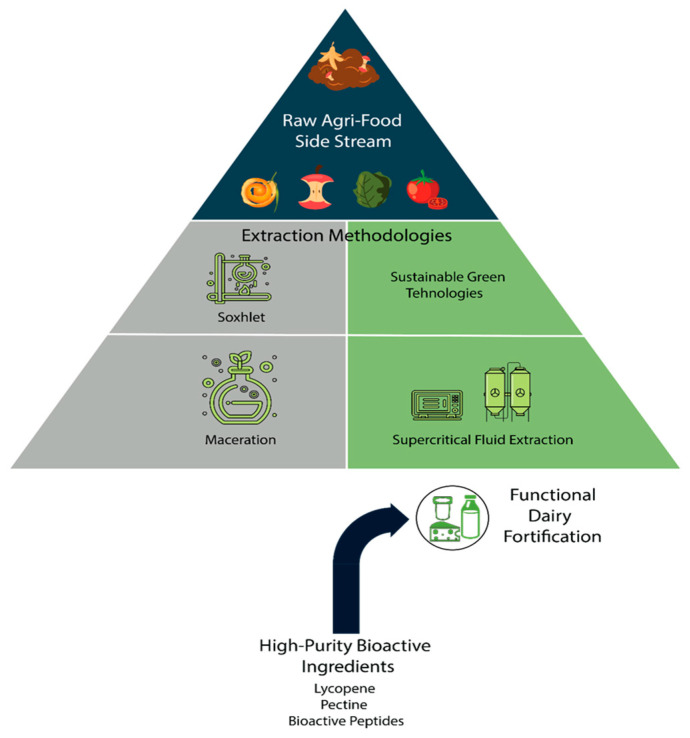
The valorisation of agri-food side streams.

The transformation of such bulk residues into high-value functional ingredients is governed by a hierarchical valorisation process (Figure 3), in which raw side streams are progressively refined through extraction technologies—ranging from conventional methods to advanced green approaches [31,32]. This transition is critical, as it determines not only extraction efficiency but also the stability and applicability of the recovered compounds in dairy matrices.

Figure 3 emphasises the hierarchical transformation of low-value biomass into high-value functional ingredients, underlining the critical role of extraction technologies in determining both yield and functional applicability.

Cereal-based side streams, particularly bran and husks, complement plant-derived residues by contributing lignocellulosic fibres and phenolic acids with both nutritional and structural functionality [33]. Their incorporation into dairy systems, such as processed cheese formulations, has demonstrated the ability to enhance fibre content and improve textural properties without compromising sensory acceptability [34]. These findings highlight their potential as multifunctional ingredients that simultaneously address nutritional enrichment and structural optimisation.

A comprehensive overview of the principal by-product sources, their dominant bioactive fractions, and their specific technological roles in dairy applications is presented in Table 1. The comparison highlights that while plant-derived residues provide diverse functional compounds, their applicability in dairy systems is often constrained by sensory and stability limitations, reinforcing the need for controlled formulation strategies.

A comprehensive overview of the principal by-product sources, their dominant bioactive fractions, and their specific technological roles in dairy applications is presented in Table 1. The comparison highlights that while plant-derived residues provide diverse functional compounds, their applicability in dairy systems is often constrained by sensory and stability limitations, reinforcing the need for controlled formulation strategies. However, the heterogeneity of these by-products in terms of composition and particle size remains a significant challenge, requiring more explicit standardisation strategies to ensure reproducible functional performance in dairy applications. These strategies may include batch classification based on compositional profiling, the homogenisation of particle-size distribution through controlled milling or sieving, and the standardisation of key pre-treatment and extraction variables, such as moisture content, solid-to-liquid ratio, temperature, and processing time. Such measures are particularly important for reducing batch-to-batch variability, improving comparability across studies, and ensuring more consistent techno-functional behaviour during incorporation into dairy matrices.

### 4.2. Intrinsic Valorisation: The Role of Dairy Processing Effluents

In addition to plant-derived residues, the dairy industry itself generates significant by-products, with whey representing the most prominent example. Traditionally considered a waste stream, whey is now recognised as a valuable resource due to its high content of lactose, proteins, and bioactive peptides [43].

Advances in membrane-based separation technologies have enabled the efficient fractionation of whey into high-value components, which can be re-integrated into dairy formulations to enhance nutritional value and functional performance [44]. These recovered fractions can modulate textural properties, improve protein content, and contribute to the development of novel dairy-based functional products.

From a circular economy perspective, the internal reutilisation of dairy processing effluents represents a closed-loop strategy that reduces waste generation while maximising resource efficiency [26]. This approach aligns with the broader nutrient recovery framework illustrated in Figure 1 and reinforces the role of the dairy sector as both a generator and a valoriser of functional by-products.

However, despite these advances, the economic feasibility and scalability of these recovery processes remain highly dependent on processing costs and market acceptance, representing a key limitation for widespread industrial adoption.

## 5. Phytochemical Profiles and Bioactive Potential of Agri-Food Derivatives

The functional and health-promoting potential of agri-food by-products is intrinsically linked to their complex phytochemical composition, encompassing high-molecular-weight polysaccharides, polyphenolic compounds, and lipophilic micronutrients [25,45]. When incorporated into dairy systems, these bioactive constituents do not act as inert additives but actively participate in physicochemical and biochemical interactions that influence both nutrient bioavailability and matrix stability [46]. These interactions are highly dependent on compound structure, concentration, and processing conditions, highlighting the importance of a mechanistic understanding of their behaviour in dairy environments. Importantly, the functional relevance of these bioactive fractions should not be considered independently of dose-dependent effects, since the same compound may exert beneficial technological or health-related effects at moderate inclusion levels but become detrimental to texture, flavour, or matrix stability when added in excess. In addition, the bioaccessibility and potential bioavailability of phytochemicals in dairy systems may be modified by their interactions with milk proteins and fat globules, which can either improve protection during processing and digestion or reduce the freely available fraction of the compound [47]. To provide an integrated overview of the main bioactive fractions discussed in this section, Table 2 summarises their predominant sources, primary mechanisms of action within dairy matrices, technological and health-related impacts, and the main limitations associated with their application. This comparative framework supports the discussion developed in the following subsections.

### 5.1. Dietary Fibres and Non-Starch Polysaccharides

Agri-food residues, particularly those derived from pome fruits and cereal processing, represent a significant source of dietary fibres, including pectin, cellulose, and hemicellulose [28,29]. Within dairy systems, these compounds function as natural hydrocolloids, contributing to water-binding capacity and structural reinforcement.

Beyond their technological role, dietary fibres exhibit prebiotic properties, supporting the growth and metabolic activity of probiotic strains such as *Lactobacillus* spp. [53]. Their incorporation into fermented dairy products leads to reduced syneresis and improved textural stability, primarily through the formation of a three-dimensional network that interacts with casein micelles. In yogurt-type systems, this effect may be accompanied by improved water-holding capacity, increased colloidal stability, and a denser gel microstructure, as shown for high-methoxyl pectin recovered from grape pomace and incorporated into low-fat synbiotic yogurt [54]. However, the functionality of fibre-rich ingredients remains strongly influenced by particle size, degree of solubility, and level of incorporation, all of which may affect mouthfeel, product homogeneity, and gel behaviour. Accordingly, the contribution of fibre-rich fractions to dairy functionality is not simply compositional, but also depends on achieving an appropriate balance between water immobilisation, structural reinforcement, and sensory acceptability.

### 5.2. Polyphenolic Compounds: Antioxidant and Structural Roles

Polyphenolic compounds, including phenolic acids and flavonoids, are among the most extensively studied bioactive constituents of agri-food by-products, particularly those derived from grape seeds and citrus residues [42]. Their primary functional role in dairy systems is associated with their strong antioxidant capacity, which enables the inhibition of lipid oxidation in high-fat matrices such as cheese and butter, thereby enhancing shelf-life and preserving sensory quality [22].

In addition to their antioxidant effects, polyphenols interact directly with milk proteins through hydrogen bonding, hydrophobic interactions, and other non-covalent associations. These interactions can significantly modify the structural and rheological properties of dairy systems, influencing gel formation, curd firmness, and protein aggregation behaviour [20]. At the same time, protein–polyphenol binding may alter the bioaccessibility and potential bioavailability of these compounds, since the bound fraction may not behave in the same way as the free compound during digestion. As discussed by Adinepour et al. [47], interactions with caseins and whey proteins may either protect bioactive compounds against degradation during processing and gastrointestinal transit or reduce their immediately measurable antioxidant activity and free concentration in the product. For this reason, the expected health-related effects of polyphenol enrichment should be interpreted not only in relation to the intrinsic bioactivity of the added compounds, but also to their behaviour after incorporation into the dairy matrix.

Importantly, the effects of polyphenols are often dose-dependent. Moderate incorporation may improve oxidative stability and, in some cases, contribute positively to gel structure, whereas excessive concentrations may intensify protein binding and lead to undesirable sensory attributes such as bitterness and astringency, as well as possible changes in texture and bioavailability. These considerations highlight the need for controlled incorporation strategies that take into account both functional efficacy and matrix-dependent limitations.

### 5.3. Carotenoids and Lipophilic Micronutrients

Lipophilic bioactive compounds, such as lycopene from tomato residues and β-carotene from carrot by-products, represent an important class of natural pigments and antioxidants [30]. Their incorporation into dairy products provides a dual benefit, combining natural colour enhancement with potential health-promoting effects.

Due to their lipophilic nature, carotenoids exhibit preferential partitioning within the lipid phase of dairy matrices. Milk fat globules play a crucial role as carriers, facilitating the formation of mixed micelles during digestion and thereby enhancing intestinal absorption and overall bioaccessibility [29]. In this respect, dairy systems may act not only as delivery matrices but also as modulators of carotenoid bioaccessibility, especially when appropriate stabilisation or encapsulation strategies are used [47]. However, these effects remain dependent on the concentration of the incorporated compound, the fat content of the product, and the extent to which carotenoids are protected from oxidation during processing and storage.

Nevertheless, these compounds are highly sensitive to oxidative degradation, light exposure, and thermal processing, which may limit their stability during storage and processing. As with other phytochemicals, the technological and nutritional value of carotenoid enrichment is therefore dose-dependent: low to moderate levels may improve pigmentation and antioxidant potential, whereas excessive addition or insufficient protection may result in instability, uneven distribution, or reduced product quality. Overall, the successful application of these bioactive compounds in dairy systems depends on achieving an appropriate balance between functional enhancement, bioaccessibility, and technological or sensory constraints, thereby highlighting the need for optimised formulation strategies.

## 6. Extraction and Recovery of Value-Added Compounds

The valorisation of agri-food by-products into functional ingredients requires extraction and recovery strategies that balance efficiency, selectivity, and preservation of bioactivity. In this context, the primary challenge is not only to maximise yield but to maintain the structural integrity of thermolabile compounds, such as polyphenols, carotenoids, and vitamins, which are highly susceptible to degradation under thermal, oxidative, and pH-induced stress conditions [55,56].

Consequently, modern extraction should be framed as a combined recovery–stabilisation problem, in which the optimal process maximises the recovery of intact bioactive molecules per unit of solvent, energy, and time, while remaining compatible with food-grade requirements and industrial scalability. This perspective is particularly relevant for dairy applications, where extract stability directly influences functionality and shelf-life.

**Figure 4:**
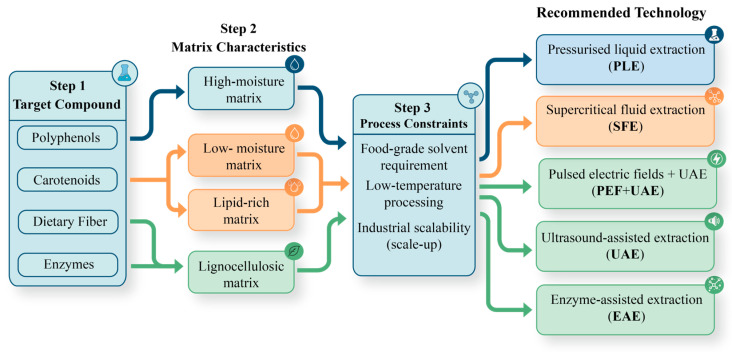
Conceptual decision framework for selecting extraction strategies based on target compound characteristics, matrix properties, and process constraints.

**Figure 5:**
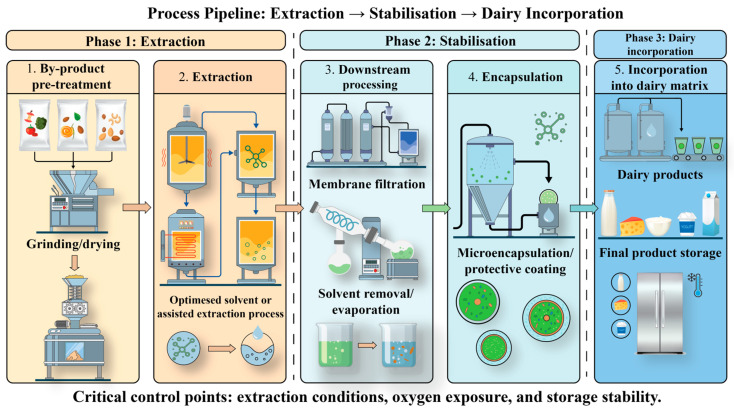
Integrated process scheme linking agri-food by-product pre-treatment, extraction, downstream recovery, stabilisation, and incorporation into dairy systems.

A conceptual decision framework for selecting appropriate extraction strategies based on compound class, matrix characteristics, and processing constraints is presented in Figure 4, while Figure 5 illustrates the integrated process pathway from by-product pre-treatment to extraction, stabilisation, and incorporation into dairy systems.

Together, these frameworks highlight the importance of aligning extraction strategy selection with downstream processing and final product functionality in dairy systems.

### 6.1. Key Variables Governing Extraction Efficiency and Stability

Extraction efficiency is determined by the interplay between:

(i) The structural characteristics of the raw material (cell wall integrity, particle size, moisture content);
(ii) The physicochemical properties of target compounds (polarity, molecular weight, binding interactions);
(iii) Process parameters (temperature, pH, solvent system, solid-to-liquid ratio, residence time, and agitation).

Conventional techniques, including maceration, Soxhlet extraction, and hydrodistillation, remain widely used due to their operational simplicity. However, their reliance on prolonged extraction times and elevated temperatures increases the risk of degradation of thermolabile compounds and often results in lower functional quality of extracts [55,56].

### 6.2. Conventional Extraction: Efficiency Versus Stability Trade-Offs

Conventional extraction methods generally rely on organic solvents, particularly food-compatible solvents such as ethanol, often combined with thermal energy to improve mass transfer and extraction yield. Although these approaches may be effective for recovering a broad range of bioactive compounds, their application is associated with several important limitations, including possible solvent residues, high energy demand, and the partial degradation of thermolabile constituents.

A major drawback of these methods is the potential reduction in functional density, defined as the bioactivity retained per unit mass of extract, since prolonged exposure to heat may accelerate oxidation and induce structural changes in sensitive compounds. Consequently, even when extraction yield is relatively high, the biological and technological value of the final extract may be compromised. For this reason, conventional solvent-based techniques are increasingly being complemented or replaced by alternative extraction approaches that operate under milder conditions and are better suited to preserving the integrity of bioactive fractions [56,57].

### 6.3. Emerging Green Extraction Technologies: Critical Appraisal

Emerging extraction technologies have been developed to improve mass transfer, reduce solvent consumption, and preserve bioactive integrity. From a dairy-oriented perspective, however, these technologies should not be compared solely on the basis of extraction yield, but also on their ability to generate fractions that remain stable, standardisable, and compatible with subsequent incorporation into dairy matrices. Accordingly, their relevance should be assessed in relation to extract stability, scalability, and compatibility with downstream food applications, rather than extraction efficiency alone. In the context of dairy systems, it is important to distinguish between studies focused exclusively on the upstream recovery of bioactive compounds and those that extend to their direct incorporation into dairy products. While most of the literature in this field still concerns extraction-stage optimisation, a smaller number of studies have already demonstrated the feasibility of applying recovered fractions in yoghurt-based systems [54].

#### 6.3.1. Deep Eutectic Solvents (DES/NADES)

DES and NADES systems offer tunable solvent properties and reduced toxicity, making them attractive alternatives to conventional organic solvents [58,59]. Their application in extracting phenolic compounds from diverse matrices (e.g., onion, olive, tomato residues) demonstrates high versatility [60].

However, their high viscosity, challenges in solvent removal, and limited industrial standardisation remain significant barriers to large-scale application [61,62].

#### 6.3.2. Ultrasound-Assisted Extraction (UAE)

UAE enhances extraction through acoustic cavitation, thereby improving solvent penetration and mass transfer while reducing extraction time and temperature. Its effectiveness depends strongly on matrix properties and process parameters such as frequency, amplitude, and treatment intensity. In many studies, UAE remains an upstream recovery tool used to obtain phenolic- or polysaccharide-rich fractions from agri-food residues, with subsequent application in foods only inferred [63]. Nevertheless, direct dairy-oriented evidence is beginning to emerge. For example, grape pomace high-methoxyl pectin was recovered using a sequential ultrasound-microwave extraction process and subsequently incorporated into low-fat synbiotic set yoghurts [54]. In that study, the extracted pectin improved probiotic viability, reduced syneresis, enhanced colloidal stability, promoted a denser gel microstructure, and improved rheological performance compared with pectin-free yoghurt, thereby demonstrating that the extraction stage and the final dairy functionality can be meaningfully linked within a single valorisation chain. However, as with UAE in general, excessive sonication and insufficient control of process conditions may still compromise extract quality through oxidative effects, which underscores the need for careful optimisation before dairy implementation.

#### 6.3.3. Microwave-Assisted Extraction (MAE)

MAE accelerates extraction through rapid heating of polar molecules via dipole rotation and ionic conduction, which substantially reduces extraction time relative to conventional methods [64,65]. Although many published examples focus on the efficient recovery of phenolics or other bioactives from plant residues, direct dairy validation remains limited. An important exception is the previously mentioned grape pomace pectin study, in which microwave treatment was combined sequentially with ultrasound to recover a pectin fraction later used successfully in synbiotic yoghurt formulation. This example illustrates that MAE-related strategies can be relevant to dairy applications when the recovered ingredient is subsequently evaluated in the target matrix rather than only characterised at the extraction stage.

#### 6.3.4. Supercritical Fluid Extraction (SFE)

SFE, typically using supercritical CO_2_, enables selective extraction under controlled pressure and temperature conditions, offering solvent-free extracts and improved selectivity [66,67,68,69]. High recovery of carotenoids from tomato by-products (~1200 mg/kg dry weight) demonstrates its efficiency [70]. Among the emerging extraction technologies discussed, SFE is one of the most advanced in terms of industrial implementation, particularly because it enables a high degree of process control and can be operated under automated and well-monitored conditions. However, high equipment costs and operational complexity still limit its broader industrial adoption [69].

#### 6.3.5. Other Emerging Recovery Technologies: PEF and PLE

For several other emerging technologies, including PEF and PLE, the available evidence in the present review is still dominated by upstream extraction studies [71,72]. These reports are valuable for understanding process efficiency, selectivity, and compound stability, but they do not always include validation in dairy matrices. Therefore, when discussing the relevance of such technologies for dairy applications, it is more accurate to interpret them as enabling tools for the production of bioactive-rich extracts with potential for later formulation, stabilisation, and incorporation into milk, yoghurt, cheese, or whey-based systems, rather than as technologies already validated at product level in all cited cases [73].

#### 6.3.6. Enrichment-Oriented Recovery and Stabilisation Approaches Relevant to Dairy Systems

In some cases, the transition from extraction to dairy application also involves a stabilisation step prior to incorporation. This is illustrated by the study on wild pomegranate peel, in which phenolics were first recovered by ethanol/water maceration, then microencapsulated by lyophilisation, and finally used for yoghurt enrichment. The yoghurt containing 2% lyophilised microencapsulated phenolic extract showed the best sensory acceptability and exhibited significantly higher phenolic content and antioxidant activity, while retaining high levels of phenolics, flavonoids, and antioxidant properties during 14 days of refrigerated storage. This type of study is particularly relevant to the dairy sector because it moves beyond extraction yield alone and demonstrates that extract stabilisation and matrix compatibility are decisive for practical application [73]. 

A comparative overview of representative extraction approaches, operating conditions, reported yields, and their relevance to dairy applications is presented in Table 3. To improve clarity, the table distinguishes between studies focused primarily on upstream recovery of value-added compounds and those that extend to direct validation in dairy products.

As illustrated in Table 3, most of the cited studies focus on the upstream recovery of bioactive fractions from agri-food by-products, whereas direct validation in dairy matrices remains comparatively limited. This distinction is important because extraction efficiency alone does not guarantee successful application in dairy systems, where matrix interactions, storage stability, and sensory acceptability must also be considered. Notably, the available yoghurt-based examples demonstrate that when extraction is coupled with appropriate stabilisation and formulation strategies, recovered by-product fractions can contribute meaningfully to functional dairy product development. Accordingly, the practical relevance of extraction technologies for dairy applications depends not only on compound recovery, but also on the downstream operations required to concentrate, standardise, stabilise, and deliver these fractions in a form suitable for food formulation.

### 6.4. Downstream Recovery: Membrane-Based Concentration, Fractionation, and Stabilisation

In this sense, downstream recovery and stabilisation should be viewed as continuations of the extraction stage rather than as independent technical steps, since they determine whether the recovered phytochemicals can be converted into reproducible and functionally relevant dairy ingredients.

Membrane technologies, including microfiltration, ultrafiltration, and nanofiltration, play a critical role in downstream processing by enabling the clarification, concentration, and partial fractionation of phytochemical-rich extracts under non-thermal conditions [12]. Beyond simple volume reduction, these operations can be used to remove suspended material, enrich target fractions, and reduce the need for high-temperature concentration steps that may compromise thermolabile compounds. In this way, membrane-based recovery contributes not only to extract purification, but also to the preservation of functional integrity prior to formulation.

This step is particularly relevant for dairy-oriented applications, where the compositional consistency and stability of the recovered fraction strongly influence its subsequent behaviour in milk-based systems [84]. By limiting oxygen exposure and avoiding severe thermal stress, membrane processing helps maintain the stability of sensitive compounds before their incorporation into dairy matrices. In addition, the controlled concentration and separation of phytochemical fractions may facilitate the preparation of more standardised ingredients, which is important for improving reproducibility, compatibility with dairy formulations, and the efficiency of subsequent stabilisation steps such as encapsulation.

### 6.5. Stabilisation Strategies: Encapsulation and Delivery Systems

Following extraction and downstream concentration, bioactive compounds remain highly susceptible to degradation during storage, processing, and digestion. For this reason, their preparation prior to incorporation into dairy matrices requires more specific stabilisation strategies than a generic reference to encapsulation alone. Depending on the physicochemical properties of the target compound, several delivery systems may be used, including spray drying, freeze-drying, extrusion gelation, coacervation, emulsification-based systems, and liposomal carriers [85,86]. These approaches differ in their suitability for hydrophilic or lipophilic compounds, in their tolerance to processing stress, and in their ability to modulate release behaviour in the final product.

Spray drying is one of the most widely applied methods because it generates powders with low water activity and good storage stability, which are convenient for transport, storage, and re-dispersion in dairy formulations [87]. Freeze-drying is more appropriate for highly heat-sensitive compounds, since it avoids the elevated temperatures associated with conventional drying. Extrusion gelation and coacervation are particularly useful when mild processing conditions and controlled release are required, while emulsification-based systems and liposomal carriers are especially relevant for lipophilic compounds such as carotenoids and certain essential fatty acids. In practical terms, these approaches are not interchangeable, and no single encapsulation technique can be considered universally optimal for all phytochemical fractions intended for dairy use. Instead, the selection of the delivery system should be guided by compound polarity, molecular structure, thermal sensitivity, and the characteristics of the target dairy matrix.

This specificity is especially important in dairy systems because unprotected bioactives may interact with milk proteins and fat globules, lose antioxidant activity, or undergo degradation during processing and storage. Encapsulation in protein- or polysaccharide-based matrices has therefore been used to improve stability, control release, and enhance bioaccessibility after incorporation into milk, yoghurt, cheese, and other dairy products [88,89,90]. Nevertheless, despite these advantages, regulatory considerations and the need for stronger in vivo validation remain important constraints for large-scale implementation [85].

## 7. Health Relevance of Recovered Phytochemicals in Dairy Systems

Recovered phytochemicals from agri-food by-products are frequently associated with antioxidant, anti-inflammatory, antimicrobial, cardiometabolic, and gut-health-related effects. However, in dairy applications, these benefits should not be inferred directly from the composition of the original plant material alone. Their actual relevance depends on whether the recovered fractions remain stable, bioaccessible, and functionally active after incorporation into milk, yoghurt, cheese, or other dairy matrices, and after exposure to processing, storage, and gastrointestinal conditions. For this reason, the health significance of by-product-derived ingredients in dairy systems must be discussed in relation to matrix-dependent transformations, dose-dependent effects, and translational feasibility, rather than as a generic nutraceutical narrative [26,47].

### 7.1. Matrix Interactions and Transformations as Determinants of Health Relevance

Once incorporated into dairy products, phytochemicals no longer behave as free compounds. Polyphenols may interact with caseins and whey proteins through hydrogen bonding, hydrophobic interactions, and other non-covalent associations, which can alter their free concentration, antioxidant behaviour, and digestion profile. As highlighted in dairy-oriented reviews, these interactions may reduce the immediately measurable antioxidant activity of the free fraction, but may also protect sensitive compounds against degradation during processing and gastrointestinal transit, so the overall effect on bioaccessibility and potential bioavailability is matrix-dependent rather than universally positive or negative [47].

Lipophilic phytochemicals follow a different pattern. Carotenoids may benefit from incorporation into fat-containing dairy matrices because milk fat globules can support mixed-micelle formation during digestion and thereby improve bioaccessibility. Nevertheless, this potential advantage remains conditional on oxidative protection, homogeneous distribution, and appropriate formulation, since carotenoids are highly sensitive to oxygen, light, and heat. Accordingly, dairy products may function not only as carriers, but also as modulators of the biological relevance of recovered phytochemicals.

### 7.2. Dose-Dependent Effects in Fortified Dairy Systems

The health and functional relevance of these ingredients is also dose-dependent. Moderate inclusion levels may improve oxidative stability, probiotic performance, water-holding capacity, or texture, whereas excessive enrichment can intensify bitterness, astringency, darkening, texture defects, or phase instability. Therefore, a higher phytochemical load does not automatically result in a better dairy product.

This is illustrated by yoghurt-based applications. In low-fat synbiotic yoghurt, high-methoxyl pectin recovered from grape pomace improved probiotic viability, reduced syneresis, and enhanced gel structure, but the final formulation still required optimisation, with 1.88% identified as the most suitable inclusion level overall [54]. Likewise, in yoghurt enriched with lyophilised microencapsulated phenolic extract from wild pomegranate peel, 2% was selected as the optimal dose because it provided improved phenolic retention and antioxidant activity without unacceptable sensory deterioration [72]. These studies show that the practical value of by-product-derived ingredients depends on balancing enrichment with matrix compatibility and consumer acceptability.

### 7.3. From Generic Health Claims to Matrix-Validated Functionality

Many health claims associated with agri-food by-products originate from in vitro screening, compositional profiling, or non-dairy applications. Such evidence remains useful for identifying promising compounds, but it should not be transferred uncritically to dairy foods. In dairy systems, the more relevant question is whether the compound remains available in a biologically meaningful form after protein binding, fermentation, storage, and digestion.

For this reason, the most informative evidence comes from studies that evaluate both product performance and matrix behaviour. In practice, a stronger dairy-oriented interpretation should link health relevance to parameters such as oxidative stability within the product, retention of phenolics during storage, support of probiotic viability, and maintenance of acceptable sensory and textural quality. A critical review should therefore prioritise matrix-validated functionality over generalized nutraceutical extrapolation [26].

### 7.4. Critical Framework for Interpreting Health Relevance in Dairy Systems

Taken together, these observations indicate that recovered phytochemicals should not be evaluated in dairy matrices solely as sources of antioxidant or health-promoting compounds. Their actual value depends on matrix-dependent transformations, protein binding, oxidative stability, dose selection, and the preservation of bioaccessibility after incorporation into the final product. This framework is summarised in Table 4.

### 7.5. Regulatory and Industrial Translation of Novel Dairy Ingredients from Agri-Food By-Products

Beyond scientific proof-of-concept, the use of by-product-derived ingredients in dairy foods also depends on regulatory acceptability and industrial readiness. In the European Union, ingredients that qualify as novel foods require pre-market authorisation, and authorised products are then included in the Union list under specified conditions of use and labelling requirements. The European Commission also maintains a Novel Food status catalogue as a non-binding support tool for assessing regulatory status.

In the United States, ingredients intended for food use generally need either food additive approval or a GRAS basis supported by appropriate scientific evidence under the intended conditions of use. FDA also maintains a public GRAS Notice Inventory, which provides a practical reference point for ingredient developers.

From an industrial perspective, the main benchmark is not extraction yield alone, but whether the ingredient can be standardised, stabilised, accepted sensorially, and integrated into manufacturing without compromising product quality or safety. Dairy-focused reviews emphasise that practical implementation remains constrained by compositional variability, contaminant control, sensory acceptability, cost, and the still limited number of studies that include robust safety assessment under realistic product conditions [26].

## 8. Strategic Applications of Agri-Food By-Products in Dairy Systems

The incorporation of agri-food by-products into dairy matrices has emerged as a central strategy for enhancing nutritional quality and functional performance while simultaneously addressing the environmental challenges associated with food waste. Dairy systems, characterised by their complex protein–lipid architecture and buffering capacity, provide favourable environments for stabilising plant-derived bioactive fractions and facilitating their controlled release. However, the success of such fortification strategies is governed by intricate interactions between bioactive compounds, dairy components, and processing conditions, which influence technological behaviour, sensory properties, and industrial feasibility [47].

Beyond compositional enrichment, the successful incorporation of agri-food by-products into dairy systems depends on their interactions with the protein–fat network of the final product. Polyphenols may interact with caseins and whey proteins through hydrogen bonding, hydrophobic interactions, and, in some cases, non-covalent complex formation, thereby influencing gel structure, antioxidant retention, and bioavailability. Similarly, fibre-rich fractions may enhance water-holding capacity and reduce syneresis by reinforcing the gel network or increasing water immobilisation, although excessive addition may negatively affect mouthfeel and firmness [47,97]. These ingredients may also modify fermentation kinetics by altering substrate availability, buffering conditions, or microbial performance, with consequences for acidification rate, texture development, and final product stability. Therefore, the technological and nutritional effects of by-product incorporation should be interpreted in relation not only to composition, but also to matrix compatibility, inclusion level, and processing conditions [54].

This section provides a comparative and critically oriented discussion of the integration of agri-food by-products across major dairy categories. Particular attention is given not only to the technological synergies and functional benefits associated with these applications, but also to the matrix-dependent constraints that frequently limit their success, including dose-related effects, sensory deterioration, physicochemical instability, and incompatibility with industrial processing conditions [16,47]. By examining these trade-offs across different dairy systems, this section aims to identify the conditions under which by-product-derived ingredients can move beyond laboratory-scale promise toward practical dairy implementation [26].

### 8.1. Cheese and Coagulated Dairy Matrices

Cheese represents one of the most extensively investigated dairy substrates for by-product fortification due to its structural diversity and the biochemical transformations that occur during ripening. As reported by Difonzo et al. [16], cheese matrices can effectively entrap and stabilise sensitive phytochemicals, thereby enabling functional enrichment without necessarily compromising structural integrity. In this context, the applications discussed here refer not to undefined raw fruit or vegetable residues as such, but rather to processed by-product-derived ingredients, including powders, flours, and fibre-rich functional fractions incorporated into the cheese matrix. Representative examples include grape pomace preparations, tomato peel flour, artichoke powder, and cereal fibre ingredients applied in fresh, spreadable, and semi-hard cheeses.

These fortification strategies generally lead to significant increases in total phenolic content and antioxidant capacity [5,93,98]. In addition to their functional contribution, some by-product-derived ingredients may also modify the structural behaviour of cheese matrices. For instance, tomato peel flour added at 1–3% (*w*/*w*) has been reported to enhance firmness and cohesiveness, while fibre-rich ingredients can reinforce the protein–fat network in processed cheese systems. However, these effects are strongly formulation-dependent, and the technological benefits observed at moderate inclusion levels may be offset by undesirable changes when the dosage is increased.

This trade-off is particularly evident in the case of grape pomace incorporation into spreadable cheese, where concentrations above 3% have been associated with excessive hardness, darkening, and bitterness, ultimately reducing consumer acceptability [93,99]. Therefore, the practical use of by-product-derived ingredients in cheese should not be evaluated solely on the basis of antioxidant enrichment or compositional improvement. Rather, successful application depends on careful optimisation of the form of incorporation, inclusion level, and compatibility of the ingredient with the target cheese matrix, so as to balance functional enhancement against undesirable textural and sensory modifications such as friability, colour shifts, and off-flavours.

### 8.2. Yogurt and Fermented Dairy Products

Fermented dairy systems, particularly yoghurt, offer highly suitable matrices for functionalisation due to their short processing cycles and adaptable gel network. By-products derived from apple, grape, pumpkin peel, and black carrot pomace have been widely investigated, typically at inclusion levels ranging from 0.5% to 3.0%.

Fortification consistently increases phenolic content and antioxidant activity [92,100] while fibre-rich residues act as natural stabilisers. Apple pomace additions (0.2–1.0%) significantly reduce syneresis by improving water-holding capacity and can even accelerate fermentation by providing fermentable substrates to lactic acid bacteria.

Nonetheless, metabolic interactions between phenolic compounds and starter cultures present a critical limitation. Elevated polyphenol concentrations may inhibit bacterial growth, slowing acidification kinetics. Moreover, pigment-rich residues such as black carrot and pumpkin peel impart intense colouration, which—if not carefully controlled—may exceed consumer sensory expectations and affect mouthfeel [22,26].

### 8.3. Ice Cream and Frozen Dairy Desserts

In ice cream systems, agri-food by-products fulfil dual technological roles as functional nutrients and fat replacers. Dietary fibres from citrus residues, berry pomace, and fruit pulps act as fat mimetics, increasing viscosity, reducing ice crystal formation, and improving melting resistance [100,101,102,103].

Optimal inclusion levels generally range between 1% and 2%, beyond which excessive hardness and a “grainy” mouthfeel are frequently reported. Natural pigments derived from grape skins and berry pomace provide clean-label colouring alternatives; however, anthocyanin–protein interactions can affect colour stability and contribute to astringency, requiring careful formulation control [103]. 

### 8.4. Butter, Buttermilk, and Whey-Based Beverages

High-fat dairy matrices such as butter are particularly susceptible to lipid oxidation, making them suitable targets for natural antioxidant enrichment. Studies incorporating herbal extracts (e.g., rosemary, thyme) and citrus peel powders reported enhanced oxidative stability during refrigerated storage [104,105].

Conversely, whey-based beverages offer high-value valorisation pathways for cheese manufacturing effluents. These liquid matrices effectively deliver bioactives from beetroot peel, grape residues, and fruit extracts. However, major technological challenges persist, including sedimentation, phase separation, and the masking of earthy or bitter flavour notes [105].

### 8.5. Comparative Synthesis of By-Product Applications in Dairy Systems

To summarise the technological impacts and principal limitations across dairy matrices, a comparative synthesis is presented in Table 5, highlighting both functional advantages and the constraints influencing industrial scalability.

### 8.6. Critical Perspective on Industrial Implementation

While laboratory-scale studies consistently demonstrate functional benefits, the transition to industrial-scale applications remains constrained by formulation complexity, sensory acceptability, production costs, and process compatibility [26]. Furthermore, the lack of standardisation in by-product processing and extraction protocols, together with insufficient reporting of critical process parameters, results in highly variable functional outcomes and limits reproducibility and industrial predictability [106,107]. This challenge is further reinforced by the intrinsic heterogeneity of industrial by-product streams, for which reproducible scale-up requires greater consistency in raw material quality and availability [107]. Consequently, future research should prioritise scalable processing strategies, harmonised characterisation methodologies, and long-term stability assessment under realistic production conditions.

## 9. Advanced Valorisation Pathways: Biotechnological Innovation in Dairy Processing

The strategic evolution of the circular economy within the food sector has transitioned from simple by-product incorporation to the advanced recovery of high-value processing aids. Agri-food residues are no longer viewed merely as functional additives but as complex biological substrates that can drive microbial synthesis and enzymatic innovation. This paradigm shift, as highlighted by Clerici et al. [108], allows the dairy industry to internalise the production of essential enzymes, thereby reducing reliance on synthetic or animal-derived inputs.

### 9.1. Microbial Enzyme Synthesis: By-Products as Fermentation Platforms

The heterogeneous composition of agro-industrial sidestreams—comprising cellulose, hemicellulose, and fermentable sugars—provides an optimal environment for the cultivation of enzyme-producing microorganisms. According to Clerici et al. [108], residues such as fruit pomace, cereal husks, and lignocellulosic biomass act as dual-purpose substrates: they provide a physical scaffold for solid-state fermentation (SSF) and serve as vital carbon and nitrogen reservoirs.

Within this framework, the microbial production of proteases is of paramount importance. These enzymes are indispensable for modulating the texture and flavour of dairy products through controlled proteolysis. The efficiency of these bioprocesses is contingent upon the meticulous optimisation of fermentation parameters, including moisture content, pH, and temperature, to ensure that the resulting enzymatic extracts meet the rigorous food safety and purity standards required for dairy applications.

### 9.2. Sustainable Alternatives for Milk Coagulation: Microbial Rennet

A significant technological bottleneck in global cheese production is the fluctuating availability and ethical concerns surrounding bovine rennet. Consequently, the development of microbial milk-coagulating enzymes (MCEs) through the valorisation of agri-food by-products has gained substantial academic and industrial traction.

From a mechanistical perspective, the efficacy of an alternative MCE is defined by its κ-casein specificity, which triggers the destabilisation of the casein micelle and subsequent curd formation. However, as noted in recent literature, a critical challenge involves the “proteolytic ratio”; if the enzyme exhibits excessive non-specific proteolysis, it leads to the degradation of α- and β-caseins. This over-hydrolysis can result in significant yield losses and the accumulation of hydrophobic peptides, which impart an undesirable bitter profile to the cheese matrix. Therefore, current research efforts are focused on the targeted selection of microbial strains that, when grown on by-product substrates, yield enzymes with a high clotting-to-proteolytic ratio, comparable to traditional chymosin.

## 10. Critical Challenges and Regulatory Limitations in By-Product Valorisation

The transition from academic experimentation to industrial-scale implementation of agri-food by-products is currently impeded by a complex interplay of regulatory, technological, and socio-economic barriers. As observed by Rodrigues et al. [25], the existing global regulatory framework prioritises waste prevention and reduction rather than the systemic transformation of these streams into high-value food-grade ingredients.

### 10.1. The Legislative Gap: Purity and Safety Standards

Within the European Union (EU), the utilisation of food-derived by-products is governed by the intended final use of the product, yet specific guidance for processing residues remains sparse. Precup et al. [109] highlight a critical legislative oversight: while Commission Regulation (EC) No 1881/2006 [110], stipulates maximum levels for contaminants in finished foodstuffs, it fails to explicitly address by-products such as peels, pomace, or seeds. This ambiguity, further exacerbated by the exclusion of these materials from safety annexes in Commission Regulation (EC) No 2073/2005 [111], and (EU) 2018/62 [112], creates a significant barrier for manufacturers who must meet stringent safety requirements without a tailored legal roadmap. Consequently, as noted by Hasan et al. [27], much of the innovative research in this field remains confined to academic patents and pilot studies, with limited successful translation into the commercial dairy sector.

### 10.2. Technological Hurdles and Matrix Interference

The integration of plant-based residues into dairy matrices often triggers undesirable alterations in texture, flavour, and structural stability. Unlike synthetic counterparts, natural by-product-derived compounds exhibit high variability in their phytochemical concentration, influenced by botanical origin and processing history. Future efforts must focus on developing stabilising technologies, such as microencapsulation, to protect labile compounds and ensure consistent functional performance.

### 10.3. Consumer Perception and Marketability

Consumer acceptance represents a formidable bottleneck. Despite the inherent health benefits, products fortified with industrial residues are often viewed with scepticism regarding their safety and sensory appeal. Migliore et al. [113] argue that the industry’s primary challenge lies in raising awareness of the economic and environmental dividends of these sustainable products. To bridge this gap, producers must move beyond purely functional claims and invest in descriptive sensory evaluations to create products that align with consumer expectations for both taste and “clean-label” transparency.

### 10.4. Economic Feasibility and Supply Chain Logistics

Beyond the technical challenges, the industrial readiness of by-product valorisation is tied to the stability of the supply chain. The seasonal nature of fruit and vegetable processing creates fluctuations in the availability of raw materials, complicating the continuous production of fortified dairy systems. Furthermore, the energy-intensive nature of certain green extraction technologies may offset the environmental benefits unless the process is optimised for industrial scale-up.

### 10.5. Scale-Up Considerations for Industrial Application in Dairy Systems

The industrial translation of plant-derived by-product valorisation into dairy systems requires a broader assessment than laboratory-scale proof-of-concept alone. Dairy products are promising vehicles for the incorporation of recovered value-added compounds because they may enhance nutritional, technological, and sensory attributes; however, such fortification strategies must preserve the physical characteristics, palatability, and consumer acceptability of the final product [26]. In this context, scale-up should be considered as a multi-dimensional process involving not only extraction efficiency, but also raw material availability, process standardisation, formulation compatibility, and industrial feasibility. From the extraction perspective, recent evidence indicates that the transition from bench-scale to pilot and semi-industrial systems remains a critical bottleneck, since reactor configuration, operation mode, and energy distribution strongly influence process productivity and reproducibility [106]. In particular, flow-cell and flow-through approaches appear more suitable for larger processing volumes, whereas batch systems are generally less productive and more difficult to scale while maintaining uniform power density. Complementary semi-industrial evidence further shows that scale-up is technically feasible, but not without trade-offs: when hazelnut skin polyphenols were transferred from laboratory to semi-industrial subcritical water extraction, extract quality and bioactivity were largely preserved, whereas extraction yield decreased because of process adaptations such as a lower solid-to-liquid ratio and different heating conditions [107].

Besides extraction performance, the successful implementation of recovered bioactives in dairy matrices depends on maintaining their stability and functionality throughout processing, storage, and consumption. Bioactive compounds may undergo degradation under oxygen, light, temperature, or harsh processing conditions, and they may also interact with milk constituents, particularly caseins and whey proteins, which can reduce their bioavailability and antioxidant activity [47]. These matrix-related constraints are especially relevant for phenolic compounds, whose affinity for milk proteins may alter both functional efficacy and final product quality. For this reason, encapsulation strategies represent an important technological tool for industrial application, as they can protect sensitive compounds during processing, storage, and transport, while reducing adverse interactions with milk components. Nevertheless, no single encapsulation approach is universally applicable, since the optimal system depends on the molecular characteristics of the bioactive ingredient and the physicochemical properties of the target dairy product. Overall, effective scale-up for dairy applications should therefore integrate extraction optimisation, downstream processing, stability-preserving formulation strategies, and quality standardisation of heterogeneous agro-industrial raw materials in order to ensure reproducible performance under industrial conditions [107,108].

## 11. Future Perspectives and Concluding Remarks

The long-term environmental and economic sustainability of the global food industry is inextricably linked to the strategic valorisation of secondary side streams. Beyond the primary focus on fruit and vegetable residues, emerging research must broaden its scope to include a more diverse array of agro-industrial effluents—such as eggshells, seafood shells, and various cereal by-products—transitioning from a disposal-centric model to one of comprehensive resource recovery. In the dairy sector, this strategy facilitates the production of value-added functional foods, where bioactive compounds and enzymes derived from food waste significantly enhance the nutritional and physicochemical profiles of products like cheese and yogurt.

The successful industrial transition toward a more resilient food system is contingent upon a cross-disciplinary collaboration between academic researchers and industry stakeholders. As highlighted by Hassoun et al. [114], the adoption of advanced Industry 4.0 technologies—including Artificial Intelligence (AI), the Internet of Things (IoT), and smart sensors—will be pivotal in managing the inherent variability of natural by-products, allowing for real-time quality prediction and process optimisation. Furthermore, to overcome the persistent challenges of ingredient stability and matrix interference, the application of nanotechnology and microencapsulation offers a transformative pathway to protect labile polyphenols and enhance their bioaccessibility within the digestive tract [115].

**Figure 6:**
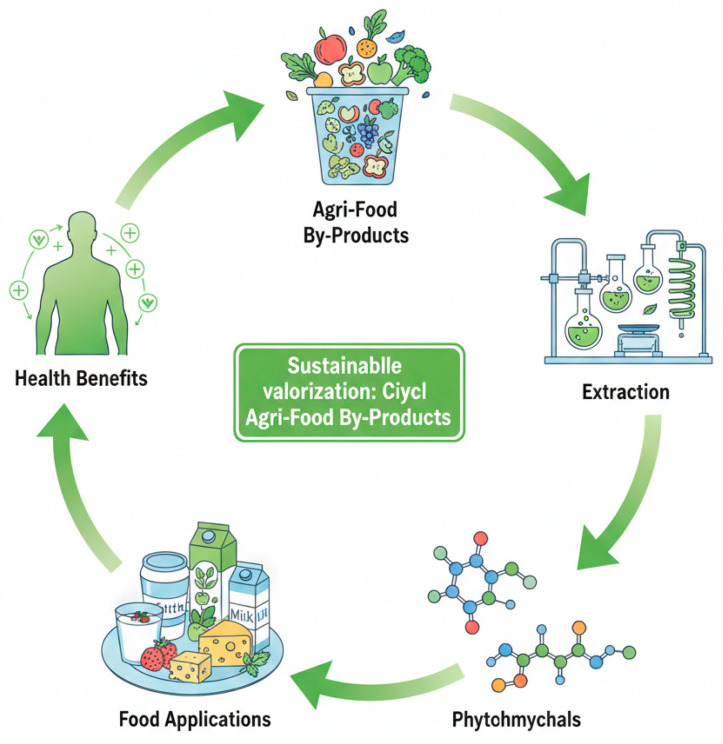
Pictorial presentation of Sustainable Valorisation of Agri-Food By-Products.

The integrated framework for this transition is synthesised in Figure 6, which illustrates the holistic cycle of sustainable valorisation. However, for this cycle to achieve true industrial readiness, future studies must incorporate rigorous Life Cycle Assessments (LCA) to ensure that the environmental gains of recovery are not offset by the energy demands of extraction processes. Moreover, as the industry moves towards commercialisation, understanding the “upcycled” market becomes essential; recent evidence suggests that consumer trust and willingness to pay are significantly influenced by transparent labelling and the effective communication of environmental dividends [116].

Ultimately, the valorisation of agri-food waste represents a transformative opportunity for the dairy sector to develop next-generation products—such as ice creams and fermented milks enriched with recovered phytochemicals—that align with both consumer health demands and global sustainability mandates. By bridging the gap between molecular innovation and consumer psychology, the dairy industry can lead the transition towards a truly circular and resource-efficient bioeconomy.

## 12. Conclusions and Strategic Roadmap

This comprehensive review elucidates that the strategic valorisation of agri-food by-products is no longer a peripheral environmental concern but a core technological imperative for the modern dairy industry. The evidence synthesised across this study confirms that residues from fruit, vegetable, and cereal processing represent a sophisticated reservoir of bioactive constituents—ranging from lignocellulosic fibres to complex polyphenolic fractions and microbial enzymes. When systematically integrated into dairy matrices such as cheese, yoghurt, and ice cream, these secondary raw materials facilitate a multi-dimensional enhancement of the final product, improving antioxidant capacity, textural resilience, and nutritional bioavailability.

The paradigm shift “From Waste to Wealth” requires moving beyond laboratory-scale successes to address the systemic bottlenecks identified in this review:

- Technological precision: Future applications must transition towards advanced stabilisation techniques, such as nano-encapsulation, to mitigate sensory trade-offs (e.g., bitterness or graininess) while ensuring the functional integrity of labile phytochemicals during the rigorous thermal processing of dairy items [25].
- Regulatory harmonisation: A significant barrier remains the “legislative gap” highlighted by [109,113], where the lack of specific safety annexes for food-grade by-products creates industrial uncertainty. Establishing clear purity and contaminant standards (specifically for peels and pomace) is essential for commercial translation.
- Digital and circular integration: As suggested by Hassoun et al. [114], the adoption of Industry 4.0 tools—including AI-driven quality monitoring and IoT-enabled supply chains—will be the catalyst for managing the inherent seasonal variability of agri-food biomass, ensuring a consistent and safe supply for large-scale dairy fortification.

In conclusion, the valorisation of agri-food waste offers a transformative opportunity to strengthen the resilience and competitiveness of the global food system. By aligning molecular innovation with circular economy principles and consumer “clean-label” expectations, the dairy sector can lead the transition toward a resource-efficient future. This review serves as a call to action for cross-disciplinary collaboration between academia, policymakers, and industry stakeholders to establish a sustainable bioeconomy where industrial side streams are permanently reclassified as valuable assets.

## Figures and Tables

**Table 1:** Integrated valorisation of agri-food by-products: linking bioactive composition, specific extraction/fractionation techniques, technological functionality, limitations, and industrial feasibility in dairy applications.

By-Product Source	Predominant Functional Fraction	Specific Green Extraction/ Fractionation Technique	Other Valorisation Approach	Dairy Application	Technological Function	Limitations/ Constraints	Industrial Feasibility	References
Pome fruits (apple)	Pectin, phenolic acids, dietary fibre	Pectin extraction; phenolic recovery by green extraction techniques *	Drying; micronisation	Yoghurt, fermented milks	Syneresis reduction; viscosity enhancement; prebiotic effect	Potential astringency; colour modification; compositional variability	High	[28,35]
Grape (*Vitis vinifera*) by-products	Proanthocyanidins, seed oils	Cold pressing; solvent-free oil recovery; polyphenol recovery by green extraction techniques *	Drying/stabilisation of pomace fractions	Ripened cheeses	Antioxidant activity; oxidative stability; lipid protection	Bitterness; dark colour; protein–polyphenol interactions affecting texture	Medium–High	[36]
Tomato (solanaceous residues)	Lycopene, carotenoids	Oleoresin extraction; carotenoid extraction *	Concentration/stabilisation of pigment-rich fractions	Cheese, functional spreads	Natural pigmentation; antioxidant activity	Colour instability; sensitivity of carotenoids to oxidation and light	Medium	[37]
Citrus by-products	Essential oils, flavanones, pectin	Cold pressing; microwave-assisted extraction (MAE)	Pectin-rich fraction recovery	Flavoured cheese, yoghurt	Antimicrobial activity; flavour enhancement; stabilising effect	Strong flavour profile; volatility of essential oils; potential bitterness	Medium	[38,39]
Cereal side streams (bran, husks)	Dietary fibre, ferulic acid	No specific extraction step in some applications; recovery of bound phenolics may involve green extraction techniques *	Micronisation; fermentation	Processed cheese	Structural reinforcement; fibre enrichment; improved water retention	Coarse texture; negative impact on mouthfeel at high inclusion levels	High	[33,40]
Cheese whey	Whey proteins, bioactive peptides, lactose	Membrane fractionation (UF/NF)	Concentration and recovery of functional fractions	Fortified dairy beverages	Nutritional enrichment; protein recovery; functional stability	Processing cost; susceptibility to microbial spoilage; stability issues	High	[41,42]

Note: * Where the cited study did not report a single clearly defined extraction step, the main recovery, fractionation, or valorisation approach is indicated.

**Table 2:** Comparative Analysis of Bioactive Fractions and their Impact on Dairy Matrices.

Bioactive Category	Predominant Sources	Primary Mechanism in Dairy Matrix	Technological/ Health Impact	Limitations/Constraints	References
Soluble/Insoluble fibres	Apple pomace, cereal husks	Water-binding; casein network interaction	Reduced syneresis; prebiotic effect; improved texture	Particle size effects; potential impact on mouthfeel	[48,49]
Polyphenols (flavonoids)	Grape seeds, citrus peels	Radical scavenging; protein–polyphenol interactions	Oxidative stability; improved curd structure; shelf-life extension	Bitterness; astringency; protein binding affecting bioavailability	[50,51]
Carotenoids (lycopene, β-carotene)	Tomato peels, carrot pomace	Lipid-phase incorporation; micelle formation	Natural pigmentation; enhanced bioaccessibility; antioxidant effect	Oxidative instability; sensitivity to light and heat	[33,40]
Organic acids	Citrus by-products	pH modulation	Antimicrobial activity; extended shelf-life	Over-acidification; potential sensory impact	[44]
Plant-derived enzymes	Proteases from plant residues	Casein hydrolysis	Alternative to animal rennet; sustainable processing	Variability in activity; process control challenges	[52]

**Table 3:** Representative studies on extraction and recovery of value-added compounds from agri-food by-products and their relevance to dairy applications.

By-Product	Technique (Key Conditions)	Target Compounds	Reported Yield/Concentration	Relevance to Dairy Applications	References
Rice bran	Soxhlet; subcritical CO_2_	Oils, tocopherols, tocotrienols	22% (Soxhlet); 13–14.5% (CO_2_)	Upstream extraction study; no direct dairy validation reported in this example	[74]
Citrus peels	Hydrodistillation vs. MAE	Essential oils	~1.7–1.8% yield	Upstream extraction study; potential relevance for flavoured dairy systems	[38,39]
Tomato waste	UAE	Lycopene, β-carotene	76.87 mg/kg dw	Upstream extraction study; potential relevance for pigment and antioxidant fortification in dairy products	[75]
Grape pomace	MAE/UAE	Polyphenols, anthocyanins	~6.68 mg GAE/g dw	Upstream extraction study; no direct dairy validation reported in this example	[76,77]
Kiwi pomace	MAE	TPC, TFC, ascorbic acid	4.8 mg GAE/g	Upstream extraction study; no direct dairy validation reported in this example	[78,79]
Banana peels	Acid extraction	Pectin	17.05%	Upstream extraction study; indirect relevance to dairy structuring applications	[80]
Blueberry by-products	PEF	Anthocyanins	223.13 mg/L	Upstream extraction study; no direct dairy validation reported in this example	[81]
Tomato peels	SFE	Lycopene	~1200 mg/kg dw	Upstream extraction study; potential relevance for fat-rich dairy matrices	[9,70]
Capsicum waste	SFE	Phenolics, carotenoids	~9–10% yield	Upstream extraction study; no direct dairy validation reported in this example	[82]
Pomegranate peel	PLE	Phenolics	194.96 mg/100 g	Upstream extraction study; no direct dairy validation reported in this example	[83]
Grape pomace	Sequential ultrasound-microwave extraction	High-methoxyl pectin	Extracted HMP used at 0.5–2% in low-fat synbiotic set yogurt; optimum formulation at 1.88% HMP	Direct dairy application (synbiotic yogurt)	[54]
Wild pomegranate peel	Ethanol/water maceration + lyophilization-based microencapsulation	Phenolic extract powder	2% microencapsulated phenolic extract selected as optimal for yoghurt enrichment; high retention of phenolics and antioxidant activity during storage	Extraction + stabilisation + direct dairy application (yoghurt)	[73]

**Table 4:** Critical framework for interpreting the health relevance of by-product-derived ingredients in dairy systems.

By-Product- Derived Ingredient	Dairy Matrix/ Application Form	Main Matrix Transformation or Interaction	Health Relevance in Dairy Context	Main Practical Limitation	References
Grape pomace polyphenol- and fibre-rich fractions	Yoghurt, cheese	Protein–polyphenol interactions; possible effects on oxidative stability and texture	May improve antioxidant potential and selected functional properties depending on dose	Bitterness, darkening, astringency, sensory penalties at high levels	[18,91,92,93]
Grape pomace high-methoxyl pectin	Low-fat synbiotic yoghurt	Gel network reinforcement; improved water immobilisation; lower syneresis	Improved probiotic viability and techno-functional performance at optimised dose	Requires formulation optimisation; effect cannot be inferred from extraction alone	[54]
Pomegranate peel phenolic extract (microencapsulated)	Yoghurt	Encapsulation improves retention during storage	Higher phenolic retention and antioxidant activity with acceptable sensory profile at optimised inclusion level	Sensory tolerance and stabilisation strategy remain critical	[73,94,95]
Citrus by-product fractions (pectin, flavonoids, essential oils)	Yoghurt, kefir, cheese	Hydrocolloid effect, pH modulation, antimicrobial activity	Potential textural and preservative contribution	Strong flavour profile, bitterness, over-acidification	[38,39]
Tomato by-product carotenoid fractions	Spreadable cheese and other fat-containing dairy systems	Partitioning into lipid phase; oxidation-sensitive	Possible colour enhancement and antioxidant contribution	Requires oxidative protection and homogeneous dispersion	[30]
Apple pomace/pectin- and fibre-rich fractions	Yoghurt, cheese	Water binding; possible support of probiotic or starter performance	Improved fibre content and possible gut-health relevance	Particle size, mouthfeel, ingredient standardisation	[28,96]

**Table 5:** Comparative synthesis of agri-food by-product applications in dairy matrices.

Dairy Matrix	Key By-Products	Main Technological Effects	Principal Constraints
Cheese	Grape pomace, tomato peel, cereal fibres	Increased antioxidant stability; enhanced firmness; reinforced structure	Bitterness; excessive hardness; colour darkening
Yogurt and fermented milks	Apple pomace, pumpkin peel, black carrot pomace	Reduced syneresis; improved water-holding capacity; increased phenolics	Inhibition of fermentation at high doses; intense pigmentation
Ice cream	Citrus fibre, berry pomace	Improved melting resistance; fat replacement; increased viscosity	Grainy texture; flavour masking challenges
Butter	Citrus peel powders, herbal extracts	Enhanced oxidative stability	Herbal off-notes; dose sensitivity
Whey beverages	Beetroot peel, fruit extracts	Natural colouring; bioactive delivery	Sedimentation; physical instability

## Data Availability

No new data were created or analyzed in this study. Data sharing is not applicable to this article.
